# Dementia Caregiving, Care Recipient Health, and Financial Burdens

**DOI:** 10.1093/geroni/igaa057.183

**Published:** 2020-12-16

**Authors:** Rashmita Basu

**Affiliations:** East Carolina University, Greenville, North Carolina, United States

## Abstract

Objective: While about 75% of people with ADRD receive care informally by their family members, relatively little is known about the effect of the quality of caregiving on maintaining carerecipient’s health and financial burden of out-of-pocket (OOP) healthcare costs. The goal of this study is to examine the quality of caregiving on the out-of-pocket healthcare costs among ADRD patients and if caregiving prevents deterioration of physical health of carerecipients. Data and Sample: We used a nationally representative sample of people diagnosed with ADRD from the Aging Demographic and Memory Study, subsample of the Health and Retirement Study. The study sample includes carerecipients whose caregivers participated in the survey (N=261). Outcome measures: Primary outcomes were deterioration of carerecipients’ health (1=yes, 0=no) and annual OOP healthcare costs. The quality of caregiving is captured by if caregiving made them feel good, feel useful and fee closer to carerecipients. More than 70% caregivers reported that caregiving make them feel good or useful. About 60% of carerecipients’ physical health was maintained, and average out-of-pocket costs was $3,701/year ($0-$31,051). Multivariable logit for binary health outcome and OLS regression for OOP cost were estimated. Results: The likelihood of health deterioration was significantly lower for carerecipients whose caregivers reported that caregiving made them feel useful (AOR=5.1, 95% CI: 1.9- 14.5) and lower OOP remained significantly associated with presence of usefulness of caregiving (cost decrease, $3000 [95% CI: $6309-$918). Positive feeling of caregiving is independently associated with lower OOP cost and deterioration of physical health among ADRD patients.

